# Recruitment and Measurement Challenges and Future Solutions

**DOI:** 10.1093/geroni/igab046.592

**Published:** 2021-12-17

**Authors:** Kimberly Van Haitsma

**Affiliations:** The Pennsylvania State University, University Park, Pennsylvania, United States

## Abstract

Recruitment of residents in the EIT-4-BPSD study required that residents have evidence of at least one behavioral symptom noted by staff in the past month. Even with this inclusion criteria 25% of the sample had no behavioral symptoms at baseline based on the Cornell Scale for Depression in Dementia, the Cohen-Mansfield Agitation Inventory, the Resistance to Care Scale, Pain in Advanced Dementia Scale, and Quality of Life in Late-stage Dementia Scale. Further, settings had good quality of care. Challenges to recruitment included the lack of willingness of residents with significant behavioral symptoms to assent to participate and legally authorized representatives to consent. Challenges to measurement included recall by staff and assumptions that behavioral symptoms were normal, short observation periods, and resident inability to provide input. Future solutions include some revisions in measures that will be described, longer assessment periods, and elimination of requirements that make recruitment of this population difficult.

